# “Double whammy”: a rapid review of rural vs urban psychosocial cancer experiences and telehealth service in five countries during the COVID-19 pandemic

**DOI:** 10.7717/peerj.14382

**Published:** 2022-11-22

**Authors:** Marisa Barnes, Kylie Rice, Clara Murray, Einar Thorsteinsson

**Affiliations:** School of Psychology, University of New England, Armidale, New South Wales, Australia

**Keywords:** Cancer, COVID-19, Pandemic, distress, Psycho-oncology, Psychosocial, Rural, Telehealth, Wellbeing, Mental health

## Abstract

**Background:**

Cancer is a long-term condition with biopsychosocial components. People with cancer living in rural areas can have poorer treatment outcomes and higher rates of unmet psychosocial needs than those in urban areas. Cancer, as opposed to other chronic conditions, poses a unique challenge in this current COVID-19 pandemic context, given immunocompromised states of patients and long-term survivor treatment effects. The disaggregated impact of psychosocial issues potentiated by the pandemic on rural *vs*. urban cancer populations is yet to be quantified. This rapid review investigates whether (i) people with cancer are experiencing pandemic-related psychosocial impacts, (ii) these impacts are equivalent in urban and rural locations, and (iii) whether the rapid uptake of telehealth mitigates or reinforces any identified impacts.

**Method:**

A rapid review was conducted for literature published between December 2019 and 13 August 2021.

**Results:**

Fifteen papers were included, incorporating evidence from five countries. The available literature suggests people affected by cancer living in rural areas are evidencing disproportionate psychosocial impacts of COVID-19, compounding cancer experiences. Despite its widespread and necessary use during the pandemic, telehealth was identified as an additional challenge for rural people with cancer.

**Conclusions:**

Clinicians working with rural people affected by cancer should ensure recognition of the greater risks of psychosocial concerns in their rural patients, and reduced access to health services. Whilst telehealth and other remote technologies are useful and necessary in this pandemic era, clinicians should consider whether its use benefits their rural clients or reinforces existing disparities.

## Introduction

With increased rates of survivorship, cancer is considered a long-term condition with biological, psychological and social components (Institute of Medicine, 2008). It is known that people with cancer, and those who care for them, exhibit high levels of distress and other mental health complications throughout the course of their cancer journey (Recklitis & Syrjala, 2017) which often persist beyond completion of medical treatment (Lu et al., 2016). Psychosocial stressors (*e.g*., emotional distress, role change, adjustment difficulties, financial and employment insecurity) have been associated with poorer functional outcomes for cancer survivors, with evidence of bi-directional and cumulative effects (Institute of Medicine, 2008).

From its first declaration as a pandemic by the WHO (World Health Organisation, 2020a) in March 2020, healthcare service delivery has been severely altered and impacted in every country around the world by the emergence of the COVID-19 virus. The pandemic has potentiated multiple psychosocial stressors through its impact not only on health, but also on employment, finances, access to services and social support. Evidence of a disproportionate psychosocial and medical impact of this pandemic on those with non-COVID conditions, particularly chronic conditions such as cancer, is just emerging (Boakye, Jenkins & Sharma, 2020). Across the world, the appropriate and necessary prioritization of the COVID-19 response has meant that there have been significant disruptions to the treatment, management, rehabilitation, and follow-up of other medical conditions, including the necessary care for cancer (World Health Organisation, 2020b). Moreover, irrespective of their unmet cancer care needs, research also indicates that people with cancer are at a higher risk of contracting COVID-19 itself and experiencing disproportionately higher adverse sequelae (Liang et al., 2020). Those with cancer, therefore, are a highly vulnerable group that warrant particular attention by psycho-oncology teams. Given the systemic immunosuppressive state of people with cancer (Liang et al., 2020), it follows that people with cancer may feel highly anxious about contracting COVID-19 itself, and justifiably fearful of experiencing serious (and possibly fatal) complications from the virus. Significant mental health problem prevalence, and gaps in mental health supports, for cancer populations have been observed during the pandemic response (Wang et al., 2020). There has also been some suggestion that cancer patients and survivors are at risk of fears resurfacing of prior traumatic medical experiences (Nekhlyudov et al., 2020). The pandemic has been described as “uncertainty upon uncertainty” for people with cancer, with multi-pronged psychosocial ramifications (Young et al., 2020).

Even prior to the pandemic, research indicated that anxiety reduced the quality of life for people with cancer and negatively impacted treatment compliance (Greer et al., 2008). A self-perpetuating cycle of pandemic-induced anxiety and risk-mitigating behavioural changes (*e.g*., social avoidance), coupled with strategies designed to reduce virus spread such as physical distancing and isolation, seems likely to occur. The immune-compromised state of people with cancer, and the existence of late effects of cancer treatments in cancer survivors, such as reduced lung function (Carver et al., 2007), compound these challenges in comparison to other chronic diseases. Given that behavioural strategies of avoidance and withdrawal are central maintaining factors within cognitive behavioural models of anxiety (*e.g*., Clark, 1986) and depressive disorders (*e.g*., Moorey, 2010), it is likely that cancer populations living through this pandemic are psychologically, as well as medically, vulnerable.

The psychosocial issues experienced by those with cancer are heterogeneous, and there are health and wellbeing disparities in existence amongst different cancer populations and groups (Carethers et al., 2020). An international meta-analysis identified significant health inequality between rural and urban cancer populations, specifically those living in rural areas are significantly more likely to die from cancer than their urban counterparts with social ecological mechanisms underlying this including individual, institution, community and policy level factors (Carriere et al., 2018). This is not just limited to adults, with some evidence that rural paediatric cancer patients experience poorer treatment experiences and outcomes (Tarnasky et al., 2021). A recent Australian report (NSW Parliament, 2022) outlined that, even prior to COVID-19, the limited availability of primary care and GP services in regional areas in New South Wales has meant that opportunities for early intervention are being lost, and that when individuals do eventually access medical assistance they then generally require more acute and complex care. Moreover, the disparate nature of service provision for the treatment or prevention of cancer, even prior to the challenges the pandemic threw at healthcare provision, has required rural people to engage with multiple services and providers where the risk of poor communication can result in rural people with cancer getting “lost in the system” (NSW Parliament, 2022). Limited and fragmented supportive care spread across different providers and locations is an ongoing challenge for all rural people, but ultimately places rural people with cancer at disproportionate risk of burden by psychosocial stressors, compounded by poorer access to psychosocial supports.

Defining what is “rural” differs from country to country, with varying synonyms and location-specific definitions (*e.g*., regional, remote, non-urban, non-metropolitan) utilised in the literature, and these definitions can also be conflicting leading to confusion and challenges when attempting to compare data (Bennett et al., 2019). For example, studies from Australia may conceptualise distance to the closest metropolitan centre very differently to studies from the United Kingdom. Hence, it is appropriate to take a place-based approach to understanding the psychosocial support needs of people with cancer during this pandemic, given the additional challenges COVID-19 and its management brings. For the purposes of this article, the term ‘rural’ will be used consistently throughout given the literature reviewed utilises varying terms to refer to people living outside of metropolitan centres with reduced accessibility of specialist services.

In Australia and across the world, rapid transition to remote service delivery models *via* telehealth has been necessary as a result of measures (*e.g*., physical distancing) designed to reduce transmission of COVID-19 (Thomas et al., 2020). What is becoming clear, however, is that this transition has not necessarily improved equitable access to services across disparate geographical regions. Countries with ready access to broadband and digital devices have been enabled to move more quickly into the remote service provision space, whilst other countries (and even socially disadvantaged locations within technologically-advanced countries) lacked the infrastructure, hardware and technical resources to modify their practices (Webster, 2020). For Australia, equivalent levels of distress in rural and urban cancer populations (*e.g*., Van Der Kruk et al., 2021) is not necessarily associated with equivalent service provision (NSW Parliament, 2022). There has been some research identifying disparities in cancer care and outcomes between urban and rural areas (*e.g*., Butow et al., 2012), and telehealth is recognised as an additional barrier for vulnerable populations such as Aboriginal Australians (NSW Parliament, 2022). Hence the question remains as to whether the COVID-19 era of service provision is perpetuating or mitigating these disparities.

There may be inadvertent “positives” to being rurally located in these times. For example, telehealth will likely have significantly reduced travel time associated with some health care appointments for those in rural areas. There may also be benefits of rural lifestyle in terms of COVID-19 transmission as a result of reduced density living and fewer government restrictions on social contact and travel. However, given the recent spread of the highly transmissible COVID-19 variants throughout regional Australia (NSW Health, 2021), for example, there are risks to the maintenance of the feeling of relative safety in rural communities.

There are, however, indications of wide-ranging impacts of the COVID-19 pandemic on physical, psychosocial and economic wellbeing of people with cancer, compounded by cancer treatment itself, in the context of the ‘new normal’ of social distancing and uncertainty (Jammu et al., 2021). Cancer survivors who have completed treatment are an additional necessary consideration in this pandemic, given persistent mental health issues are well-recognised within this group (Lu et al., 2016). Identifying vulnerable client groups is imperative, alongside the prioritisation of COVID-19, to ensure appropriate interventions are made available and healthcare systems can prioritise additional identified care needs when resources become available (Boakye, Jenkins & Sharma, 2020). Rapid reviews are recommended by the WHO to provide timely, high-quality evidence in order to support decision-making in health policy and systems (Tricco et al., 2017). As the World Health Organisation (2021, para. 4) argued, this will allow services to “build back better” for the future, for both cancer patients, survivors, and those who care for them.

### Aim

This rapid review aims to understand (i) if people with cancer are experiencing pandemic-related psychosocial impacts, (ii) whether these impacts are equivalent amongst people affected by cancer in urban *vs*. rural locations, and (iii) whether service delivery changes driven by the pandemic mitigate or exacerbate the challenges faced by rural people affected by cancer (*e.g*., access to specialist services), potentially resulting in compounded distress due to the cancer journey itself and the impacts of living within this pandemic era. This rapid review aims to foreground the experience of rural people with cancer in order to contribute to the understanding of the impacts of psychosocial stressors associated with COVID-19 on cancer journey experiences. Results may support the redirection of strengthened health service provision to support those with cancer and their families and carers living in rural areas. This rapid review will present the findings of the literature published to 13 August 2021, recognising that as the pandemic progresses and countries are experiencing multiple waves of the virus, the literature will evolve.

## Materials and Methods

### Search strategy

The WHO’s guidelines for rapid reviews (Tricco et al., 2017) and the recommendations of the Cochrane Group for rapid reviews during COVID-19 (Garritty et al., 2020) were utilised to inform this review. One author (MB) utilised the pre-established search strategy to search across the following databases: ProQuest and Informit; PubMed; the Semantic Scholar COVID-19 database CORD-19; and Google Scholar. Limited iterative searching from reference lists was undertaken of articles published after December 2019 on psycho-oncology during COVID-19 comparing rural and urban populations in order to identify additional relevant articles. Articles were excluded if the study did not include the COVID-19 pandemic or were solely focused on urban or non-segmented populations. Duplicate records were systematically identified and removed. Identification of eligible studies took place in two stages; selection based on study titles and abstracts, followed by detailed review of the full-text articles, utilising Covidence software (Veritas Health Innovation, 2021). Discrepancies and general queries were resolved through consensus of all authors. The search strategy was executed 8–13 August 2021 for articles available in English, utilising the following search strings in Title or Abstract “COVID-19” AND cancer AND psychosocial AND “rural OR regional OR remote”. For example, in PubMed the following search string was utilized: (((((COVID-19[Title/Abstract]) AND (psychosocial[Title/Abstract])) AND (cancer[Title/Abstract])) OR (rural[Title/Abstract])) OR (regional[Title/Abstract])) OR (remote[Title/Abstract]). Given COVID-19 was first identified in late 2019, the search was limited to literature published between December 2019 and 13 August 2021.

### Inclusion and exclusion of studies

Of the 1,451 articles identified, titles and abstracts were reviewed for inclusion as rural psycho-oncology studies during the COVID-19 pandemic published after December 2019. This date was chosen as some countries may have been beginning to gather and publish data earlier than when the WHO proclaimed a pandemic and some countries were affected earlier than others by the spread of COVID-19. Given the purpose of this rapid review was to provide clinicians with a useful synthesis of information as quickly as possible in this unprecedented pandemic context, as many sources of information and data were sought as possible. Hence criteria were deliberately designed to be broad and encompassing. Studies that did not refer specifically to the impact of the pandemic were excluded to ensure the analysis was only pandemic focused. Only studies allowing review of rural populations were included. Literature with quantitative data that did not disaggregate rural and urban were excluded.

### Analysis

One author (MB) extracted study details and data from the eligible studies. As is common in rapid reviews, the quality of the included studies was not appraised, which does not necessarily impact upon conclusion congruence of rapid and systematic reviews (Tricco et al., 2015). However, included literature was rated as either Tier 1 (peer-reviewed work, such as published cross-sectional studies) or Tier 2 (minimally peer-reviewed, such as expert opinion). Eleven articles were Tier 1 articles and four were judged as Tier 2. Of the Tier 2, two were oral presentation abstracts of cross-sectional study data and two were expert narrative opinion. Literature ratings were derived by consensus of all authors. A narrative synthesis of the results and conclusions of the eligible studies was conducted.

## Results

Based on the above selection criteria, and after duplicates were removed, the full text of 98 articles were reviewed. After reviewing article results and discussion, those that did not report rural results or did not report rural data relevant to the review aims, were removed, yielding 15 articles for inclusion in the narrative synthesis. Of the 15 articles, 10 considered psycho-oncology aspects of wellbeing, and five considered telehealth technologies. The articles originated from the USA (*n* = 7), Australia (*n* = 4), India (*n* = 2), Italy (*n* = 1), and Canada (*n* = 1). Table 1 shows that of the ten studies exploring psychosocial issues, seven analysed cross-sectional data of urban and rural populations, two analysed a rural-only population, and one was an expert opinion. Two of the cross-sectional studies included some qualitative data in a mixed methods design. Seven articles were assessed as Tier 1 and three as Tier 2. There was consensus amongst the Tier 1 and Tier 2 literature. However, of the Tier 2 articles, two were oral presentation abstracts of cross-sectional research data and one was an expert narrative opinion, for which greater caution should be utilised in its interpretation.

**Table 1:** Key descriptive characteristics of the studies included in the rapid review.

Study	Study type (Quality Level)	Study aims	Country	Location *(n)*	Measure/disorder/psychosocial factor	Relevant rural related findings and conclusions
Studies on psychosocial factors						
Boakye, Jenkins & Sharma (2020)	Expert opinion (Tier 2)	Opinion on the impact of the effects of the pandemic on head and neck cancer survivors’ wellbeing and health	USA	Not reported	Wellbeing	The impact of the pandemic on the continuing care needs of head and neck cancer survivors may be particularly disproportionate for those in rural areas given pre-existing paucity of material, care, and financial resources, as well as reduced help-seeking and mental health professional shortages. Potential solutions, such as telemedicine, may also be disproportionately effective in rural areas with resource access and impacts of reduced incomes.
Davis et al. (2021)	Mixed Methods (Tier 1)	To explore the impact of COVID-19 on rural cancer survivor access to health services, treatment and supportive care	Australia	Rural = 66 Urban = 0	Social support; health care access; health-related distress; emotional wellbeing (depression, anxiety, major worry); health care impact (testing/review/treatment delays)	59% experienced reduced social support; 46% experienced reduced access to health care providers; 44% experienced impact to supportive service access; 40% experienced increased health-related distress; 35% experienced negative impact on emotional wellbeing; 33% experienced major worry; 20% experienced medical testing or review delays; and 15% experienced delayed treatment. Older rural people experienced more negative impact in relation to access and wellbeing than younger participants.
Ferrara et al. (2021)	Cross-sectional (Tier 1)	To assess the perceived quality of life and the psychosocial impact of the various restrictive measures of COVID-19	Italy	Non-urban = 584 Urban = 185	Quality of life perception	Cancer patients living in non-urban areas demonstrated a more pessimistic perception of quality of life compared to their urban counterparts (OR = 1.40, 95% CI [1.09–3.10]) during the pandemic
Galica et al. (2021)	Mixed Methods (Tier 1)	To explore how older adults recently discharged from cancer care team were coping during COVID-19	Canada	Rural = 11 Urban = 19	Coping	Qualitative data suggested older cancer survivors appreciated the privileges of living in a rural area as insulation against negative impacts of COVID-19. However, for a disabled older rural person supportive care ceased due to the pandemic, impacting coping and independence.
Jacobs & Ellis (2021)	Cross-sectional (Tier 1)	To examine the social connectivity among Medicare beneficiaries during COVID-19	USA	Rural = 12 Urban = 20788	Social Connectedness	Individuals living in urban/metropolitan areas were less likely to feel disconnected (OR = 0.92, CI = 0.88, 0.97) than those living in in rural, less populous locations
Mama, Cardel & Schmitz (2020)	Oral Presentation Abstract (Tier 2)	To explore associations between perceived threat of COVID-19 and psychosocial distress on health-related quality of life	USA	Rural = 90	Quality of life, psychosocial distress	Rural cancer survivors perceived the threat of COVID-19 itself as low, however reported elevated psychosocial distress related to COVID-19, negatively impacting their mental but not physical health.
Daniels et al. (2021)	Oral Presentation Abstract (Tier 2)	To characterise the pandemic’s influence on social and health behaviours of rural and urban cancer patients	USA	Rural = 332 Urban = 994	Health behaviours	Urban and rural groups were not significantly different in their experiences of social interaction or feelings of loneliness. Urban patients felt they were at greater risk of contracting COVID-19 (22% vs, 14%; *p* < 0.001), and to more frequently feel their daily lives and exercise habits had been changed (86% vs. 77%; *p* < 0.001).
Singh et al. (2020)	Mixed Methods (Tier 1)	To assess the health, psychosocial, and economic impacts of the pandemic on people with chronic conditions, including cancer	India	Rural = 401 Urban = 409	Psychosocial and economic	Rural participants were disproportionately experiencing acute medical illnesses, difficulties accessing healthcare and medicine, less access to functioning health facilities, poorer treatment satisfaction, loss of employment/income and poorer nutrition than their urban counterparts.
Singh, Rai & Ishan (2021)	Cross-sectional (Tier 1)	To study the impact of COVID-19 lockdowns on utilisation of health-care services	India	Rural = 125 Urban = 86	Health service access	Rural participants had more difficulties accessing medicines than their rural counterparts (OR = 4.01, CI [2.90–5.53]). Rural participants were twice as likely as their urban counterparts to miss their follow-up appointments due to difficulties visiting the hospital
Zomerdijk et al. (2021)	Cross-sectional (Tier 1)	To identify the psychological impacts of COVID-19 on haematology patients and inform development of supportive interventions	Australia	Rural = 196 Urban = 198	Wellbeing; psychological distress; unmet supportive care needs; fear of cancer reoccurrence	Living in a rural area was associated with greater psychological distress during the pandemic (B -1.29 [−2.53, −0.05], *p* = 0.041). Unwanted variation in care for rural patients was likely heightened during the pandemic due to travel restrictions/barriers to accessing care
Studies on telehealth						
Brunelli, Fox & Langbecker (2021)	Mixed-Methods (Tier 1)	To investigate the preparedness of cancer nurses to deliver survivorship care *via* telehealth	Australia	Rural setting = 32 Metropolitan = 47	Telehealth	Nursing staff identified improvements in equity of access by reducing travel for patients and clinicians, allowing timely and more easily integrated connection between patients and clinicians, and incorporation of interventions. Telehealth can enhance quality of life, increase convenience, reduce burden, improve independence, and increase access to multidisciplinary metropolitan-based oncology. Despite positive nurse attitudes to use, uptake and use of telehealth remains low in all locations. Hence rural equity gap remains.
Jewett et al. (2021)	Retrospective (Tier 1)	Examined telehealth use across patient populations with established disparities in treatment and outcomes	USA	Rural = 1253 Urban = 9844	Telehealth	Telehealth was less likely to be used by rural cancer patients (45.3%) than urban (53.7%, *p* < 0.0001**). Findings underscore disparities in telehealth use across historically underserved populations.
Keefe et al. (2020)	Expert opinion (Tier 2)	To provide a basis for preparing for, and implementing, optimal management of cancer during the pandemic	Australia	Not reported	Telehealth	As a result of telehealth utilisation access to care may have improved for rural patients.
Patt et al. (2021)	Cross-sectional (Tier 1)	To characterise multi-stakeholder implementation, utilisation, and feedback of telemedicine	USA	Not reported	Cancer care uptake *via* telemedicine platforms	Implementation of telemedicine was limited by broadband access in rural communities. Recommendation for improved broadband access for rural areas as a policy priority.
Rariy et al. (2021)	Cross-sectional (Tier 1)	To describe a collaborative telehealth partnership model	USA	Not reported	Telehealth	Use of this telehealth model for rural patients reduced waiting times, reduced patient travel by 21,705 miles, saved 310 travel hours, saved US$7380 in travel and accommodation costs. The model utilises local hospitals for telehealth visits to ensure adequate broadband and technology capabilities.

When considering pandemic-related psychosocial issues experienced by people with cancer, all ten articles evinced a myriad of psychosocial concerns experienced by people with cancer in the context of the pandemic, ranging from worry and distress (*e.g*., Davis et al., 2021) to health access and burden (Singh et al., 2020), addressing this review’s first aim. When considering this review’s second aim of comparing the psychosocial experiences of rural *vs*. urban people with cancer, the results indicate significant disparities with less favourable outcomes for rural populations. Of the nine quantitative studies, three (two Australia, one USA) reported poorer emotional wellbeing for rural patients (Davis et al., 2021; Mama, Cardel & Schmitz, 2020; Zomerdijk et al., 2021), and this was similarly identified in the expert opinion (Boakye, Jenkins & Sharma, 2020). Davis et al. (2021) further noted older rural participants reported greater negative impacts on access and wellbeing than younger participants. Two studies (one USA and one Italy) reported reduced perceptions of quality of life for rural cancer populations (Ferrara et al., 2021; Mama, Cardel & Schmitz, 2020), and one USA study identified more social disconnectedness in those living in rural areas (Jacobs & Ellis, 2021). Three studies considered health service access, with two studies (India) evidencing significantly reduced health care access and increased care delays in those from rural areas compared to their urban counterparts during the pandemic (Singh et al., 2020; Singh, Rai & Ishan, 2021). The results from the qualitative data of one Canadian study (Galica et al., 2021) suggested whilst some supportive care access may have ceased, some older rural cancer survivors appreciated a sense of insulation against COVID-19 as a function of their geography. This protective effect of geography was also found in one USA study (Daniels et al., 2021), wherein the urban sample reported feeling more at risk of contracting COVID-19, and experienced greater daily life change, than the rural sample. Overall, however, social and health support access, and psychological distress-related symptoms were most likely to be worse in rural samples, with older people particularly impacted, during the COVID-19 pandemic.

The final aim of the review considered whether the rapid move to telehealth service delivery mitigates or reinforces the service provision inequities which in turn lead to disproportionate burden of adverse psychosocial inequities experienced by rural people with cancer. Five studies considering telehealth use during COVID-19 were reviewed. These included one Tier 2 expert opinion (Australia) and four Tier 1 studies, of which one was a mixed methods study (Australia), two were cross-sectional studies (USA) and one was a retrospective study (USA). All five articles recognised that the use of telehealth during the COVID-19 pandemic could go some way to improving health equity and bringing psychosocial and economic benefits to rural cancer populations, including reducing travel time and travel costs and increasing access to health and care providers (Brunelli, Fox & Langbecker, 2021; Jewett et al., 2021; Keefe et al., 2020; Patt et al., 2021; Rariy et al., 2021). However, all the Tier 1 studies identified challenges with telehealth that may impact upon the above benefits. Three USA studies found inadequate technology access and capabilities were problematic for rural cancer populations (Jewett et al., 2021; Patt et al., 2021; Rariy et al., 2021). Furthermore, one of the studies identified significantly reduced uptake of telehealth by rural cancer patients (Jewett et al., 2021), and an Australian study (Brunelli, Fox & Langbecker, 2021) found that whilst both rural and metropolitan healthcare providers held positive attitudes towards telehealth use, clinician uptake remained low.

## Discussion

This rapid review identified that people with cancer are experiencing a “double whammy” of COVID-19 disease risk burden and psychosocial impact. The immunocompromised state of patients and the impact of long-term treatment effects in survivors, that is not similarly experienced in other chronic conditions, makes this population in high need of consideration. This rapid review further examined the experience of rural people with cancer during the COVID-19 pandemic and found that the psychosocial wellbeing of rural people is disproportionately impacted. Moreover, strategies to mitigate pandemic impacts, such as telehealth, are not automatically adequate for rural people with cancer, with deliberate care required to ensure appropriateness for individual client circumstances. There may be some protective factors associated with insulation as result of ‘ruralness’, some of which appear linked to the limited spread of COVID-19 in rural communities, in contrast to the higher rates of transmission in urban areas predominately in the early days of the pandemic. However, the virus is now increasingly less urban-centric, and has spread quickly to regional, rural and remote communities (Bradford et al., 2021). As the virus continues to spread and governments are hoping to achieve endemic virus status, this may reduce the sense of relative safety in rural communities. Further, the healthcare resources of rural towns are minimal compared to urban centres (*e.g*., NSW Parliament, 2022). Rural hospitals have relatively reduced contingency and greater vulnerability to becoming overwhelmed with the COVID-19 burden, resulting in flow-on effects to primary health settings and impacts to continuity of care for rural people affected by cancer in addition to exacerbation of their psychosocial concerns.

### Psychosocial impacts

As per the first aim, the studies reviewed indicate that in general, people with cancer are facing pandemic-related psychosocial impacts alongside their existing cancer experiences. Secondly, the evidence suggests that rural cancer patients are a particularly vulnerable population in terms of their risk of adverse psychosocial sequalae during this pandemic, which adds an additional layer of complexity to any vulnerabilities to poorer COVID-19 health outcomes that may be inherent in people who have contracted the virus and have cancer as an underlying health condition. Moreover, the present review suggests a reoccurring theme where rural populations with cancer appear to be experiencing disproportionate psychosocial impacts of the COVID-19 pandemic compared to comparable urban populations. Rural people with cancer, more than their urban counterparts, are experiencing disproportionately increased social disconnectedness (Jacobs & Ellis, 2021), and greater psychological distress (Zomerdijk et al., 2021) in this pandemic, in conjunction with their existing cancer burden. This combination of internal psychological factors, and external social factors, unduly burdens rural people affected by cancer living through the COVID-19 pandemic with compounding psychosocial difficulties. The mutually exacerbating nature of psychosocial stressors and distress, including poor mental health (Mama, Cardel & Schmitz, 2020) and limited social supports, and the burden of the cancer experience journey itself, compounds the “double whammy” experienced by rural people with cancer in the context of COVID-19. Cancer-related, pandemic-related, and virus-related anxiety and distress are not just being experienced by rural people with cancer, with the services designed to support them also having been impacted by the pandemic. Davis et al. (2021) found that social support for this group has reduced significantly, likely associated with psychological distress. Moreover, reduced access to health care providers and supportive service access means that getting the right support for psychosocial difficulties is more challenging, especially when services cease (Galica et al., 2021), access is disrupted or delayed (Davis et al., 2021; Singh et al., 2020; Singh, Rai & Ishan, 2021), care provision is uncertain with imposed pandemic restrictions (Zomerdijk et al., 2021) and there is a shortage of mental health professionals (Boakye, Jenkins & Sharma, 2020). The heightened sense of vulnerability and intensified fears means psycho-oncology services are more necessary than ever for rural people affected by cancer, regardless of their stage of diagnosis or treatment.

Whilst the literature suggests that rural people generally appear to be disproportionately impacted by this pandemic, older people with cancer in rural areas have been identified as being more vulnerable (*e.g*., in terms of service access and overall wellbeing) than younger people (Davis et al., 2021). Clear risks of medical and supportive care ceasing (Galica et al., 2021) have been identified for this group. Despite the well-established need for psychosocial supports and intervention particularly in this unprecedented context, reduced help-seeking has been identified as an issue for rural people with cancer (Boakye, Jenkins & Sharma, 2020), which has likely compounded by negative perceptions of access and pandemic-management strategies such as lockdowns, social avoidance and isolation. The impact of the pandemic for rural people with cancer has additionally resulted in this population experiencing more pessimistic perceptions of quality of life (Ferrara et al., 2021). The long-term effects of these compound effects on health and wellbeing are yet to be seen.

### Telehealth in COVID-19

Telehealth is often touted as the solution to overcoming barriers to accessing services for rural people, and it is a welcome addition to the provision of both medical and psychosocial care. Its utilisation has been shown to improve equity of access to care (Keefe et al., 2020), reduce waiting times and travel time, distance, and costs (Rariy et al., 2021), and reduce burden by increasing independence and convenience (Brunelli, Fox & Langbecker, 2021). The rollout of telehealth in oncology supports continuity of clinical care, as well as providing an avenue for psychosocial care (Boakye, Jenkins & Sharma, 2020).

In consideration of this article’s third aim, whilst telehealth provides some benefit to some rural people with cancer, the findings of this review indicate that telehealth may also reinforce health inequities for other rural people, due to disparities in access to reliable technology and infrastructure required for effective service delivery. Even prior to the pandemic, challenges for telehealth delivery in cancer care and psycho-oncology services were recognised, including issues relating to standardisation of quality care, patient comfort with technology use, and ensuring privacy as well as emotional safety (Jhaveri et al., 2020). However, the rapid transition to telehealth during the COVID-19 pandemic may have inadvertently exacerbated existing disparities. Jewett et al. (2021) identified that despite reducing care logistics like travel time, telehealth was less likely to be used by rural patients, and relatively poorer broadband access limits the implementation of telemedicine for rural people (Patt et al., 2021). Challenges are not limited to patients either. Even when telehealth can be useful for rural cancer patients, there remains some reluctance on the part of health professionals to work *via* telehealth (Brunelli, Fox & Langbecker, 2021). The availability of valuable, practical guides for establishing quality telehealth consultations (*e.g*., Burbury et al., 2021) provide strong foundations for service providers which are beneficial regardless of provider familiarity and comfort with this form of practice. It is beyond the scope of this review to evaluate barriers to adoption of telehealth by rural people and health professionals, but clearly this is an area requiring further research in the interests of reducing health service inequities between rural and urban people with cancer.

### Clinical implications

For clinicians working with people affected by cancer, a range of clinical implications have been identified from the literature outlined in this review. Firstly, it is essential for cancer care providers to screen for a range of psychosocial issues in their clients during this pandemic. Mental health care is an important component of health care, and the incorporation of distress screening is well-recognised in comprehensive cancer care guidelines (Fradgley et al., 2019). There are many evidence-based tools available to healthcare providers depending on their profession, from very simple measures such as a distress thermometer to more complex inventories (National Comprehensive Cancer Network, 2020). However, even prior to the pandemic, distress screening and intervention was piecemeal at best, with distress going unassessed and undetected and ultimately untreated (Sanson-Fisher et al., 2000; Zucca et al., 2015; Zucca et al., 2016). The importance of distress screening during the pandemic and beyond is especially important for rural clients, and older people in particular, with the literature suggesting they may be both more vulnerable to distress and more reluctant to seek help (Davis et al., 2021). Clinicians should recognise the “double whammy” experienced by their rural patients in the context of COVID-19 as it adds to the burden of patients and their families due to the cumulative and compounding effects of psychosocial stressors.

Given that much of service provision is occurring utilising online platforms, clinicians require specific skills in identifying evidence of distress remotely. Undertaking additional continuing professional development and training in using telehealth for psychosocial intervention is recommended. There may be some rural people who may be reluctant to disclose psychosocial issues, and clinicians will benefit from proactively assessing engagement and responding to barriers to help-seeking in this population. Appropriate referral making is key, with early referral to psycho-oncology and psychosocial support services recommended.

Given the technology access issues that have been identified, clinicians should attempt to overcome these barriers by offering alternatives for accessing help in rural areas. For example, there may be capacity to offer service provision alternatives to support access to quality technology, such as local hospital or community resources to improve bandwidth or quality technology capabilities or supplementing telehealth with more reliable technologies such as telephone, which should be explored. In Australia the Government moved quickly to design Medicare telehealth items for GPs and Psychologists, and additional Psychology sessions eligible for Medicare rebates, and clinicians and professional bodies advocated for these to remain beyond the pandemic (Cavenett, 2021) to continue to mitigate some of the access gaps where this is appropriate, particularly for rural people for whom telehealth is a welcome and appropriate addition to their care.

Finally, the multidisciplinary opportunities inherent in healthcare settings remain a key resource for supporting the psychological wellbeing of rural people with cancer, and this should be emphasised in care and treatment plans from diagnosis through to survivorship. A recent NSW Parliament report finding that people in rural, regional and remote areas of Australia are often discharged from hospital with a lack of information and support (NSW Parliament, 2022), and the health system strain caused by the pandemic is only likely to have exacerbated this. Psycho-oncology care providers are well placed in assisting in the mitigation of the poorer health outcomes that were identified as a result of this lack of information and support.

### Limitations

The COVID-19 pandemic is ever-evolving, with multiple waves of virus transmission. This results in episodes of high lifestyle impact for populations, for example through geographical lockdowns and mask mandates. Hence, it is likely that disparities identified in this review will also fluctuate as the pandemic continues to advance. We further recognise that more recently published data may also provide information about the pandemic’s impacts that differ from the results presented here. Given the small numbers of studies of rural psycho-oncology generally, in the context of rural psycho-oncology and COVID-19 specifically, especially longitudinal studies, and the lack of formalised quality assessment of studies included in this review, more rigorous systematic and meta-analytic reviews are required. A further limitation of the study is the degree of heterogeneity in the definition of “rural” across countries. Broad generalisability of findings across countries is difficult. However, the review highlights themes in the experience of people living outside urban centres which may be common across disparate rural contexts.

## Conclusions

The pandemic has inarguably interrupted health care provision AND altered health care delivery for the foreseeable future and beyond. Hence the goal of “building back better”, including considering how pre-pandemic support services and resources can be best utilised in this potentially unpredictable pandemic future, should be a goal for all working in the psycho-oncology space. Specifically, the available evidence suggests that people with cancer are particularly medically and psychologically vulnerable during this pandemic, and rural people with cancer more so. Rural people with cancer are experiencing greater psychosocial distress during the pandemic than those living in urban areas. The necessary measures designed to limit the transmission of the COVID-19 virus are having a disproportionate detrimental effect on rural people with cancer, increasing social disconnectedness and reducing access to supportive resources and health care professionals. Given that cancer is a biopsychosocial experience, and the psychosocial needs of rural people with cancer are demonstrably greater than those of people from urban areas, these needs require focused clinical attention. If the psychosocial needs of rural people with cancer are not effectively addressed, treatment inadequacy and reduced functional outcomes are likely. To rectify the health inequity between rural and urban cancer patients, this review clearly points to a need to improve and invest in appropriate psychosocial support services for rural patients and their families.

The technology designed to support remote access to services is only a part solution, as the infrastructure is not consistently available at a sufficiently high quality for all rural people. There also remains some challenges in telehealth uptake by both rural patients and clinicians. Recognising and attempting to ameliorate the disproportionate impact of the COVID-19 pandemic on rural people with cancer is necessary for all clinicians providing psycho-oncology care. Strategic screening for distress as uniquely evidenced by rural people with cancer, coupled with clinical training to recognise distress *via* remote technologies when help-seeking may not be a characteristic of rural people affected by cancer, are key foundations in this process.

## Additional Information and Declarations

### Data Availability

The following information was supplied regarding data availability: As this is a rapid systematic review, there is no raw data.
